# 非小细胞肺癌血清中lncRNA HOTAIR的表达及临床意义

**DOI:** 10.3779/j.issn.1009-3419.2017.06.06

**Published:** 2017-06-20

**Authors:** 楠 谭, 莉红 李, 璐 白, 昆 赵

**Affiliations:** 710002 西安，西安市第一医院干部病房科 Cadre Ward, the First Hospital of Xi'an, Xi'an 710002, China

**Keywords:** 肺肿瘤, 长链非编码RNA, HOX转录反义RNA, 临床病理, Lung neoplasms, Long noncoding RNA, HOX transcript antisense RNA, Clinical pathology

## Abstract

**背景与目的:**

长链非编码RNA HOX转录反义RNA（HOX transcript antisense RNA, HOTAIR）在多种肿瘤中异常表达。本研究旨在探讨非小细胞肺癌（non-small cell lung cancer, NSCLC）患者血清中lncRNA HOTAIR的水平及其临床意义。

**方法:**

采用实时定量聚合酶链反应（polymerase chain reaction, PCR）方法检测64例NSCLC患者和64例正常对照血清中HOTAIR的水平，统计分析血清中HOTAIR的水平与NSCLC临床病理参数之间的关系。

**结果:**

与正常对照组相比，NSCLC患者血清中HOTAIR水平明显升高，两组之间的差异具有统计学意义（*P* < 0.01）。血清中HOTAIR水平与NSCLC患者的肿瘤大小、（tumor-node-metastasis, TNM）分期及淋巴转移有关（*P* < 0.05），而与患者的年龄、性别、吸烟、分化程度及病理类型均无明显相关（*P* > 0.05）。

**结论:**

NSCLC患者血清中HOTAIR水平明显升高，HOTAIR可能参与了NSCLC的发病过程。

## Abstract (en)

**Background and objective:**

The expression of long noncoding RNA HOX antisense RNA (HOTAIR) is abnormal in a variety of tumors. The aim of this study is to explore the serum levels and clinical significance of HOTAIR in patients with non-small cell lung cancer (NSCLC).

**Methods:**

The serum levels of HOTAIR were detected by real-time quantitative polymerase chain reaction (PCR) in 64 NSCLC patients and 64 normal controls. The relationships between the serum levels of HOTAIR and clinical pathological parameters were analyzed.

**Results:**

Compared with normal controls, the serum levels of HOTAIR in patients with NSCLC increased significantly (*P* < 0.01). The serum levels of HOTAIR were correlated with tumor size, tumor-node-metastasis (TNM) stage and lymph node metastasis (*P* < 0.05), but not with age, gender, smoking, differentiation and histology (*P* > 0.05).

**Conclusion:**

The serum levels of HOTAIR in patients with NSCLC are significantly higher, and HOTAIR may be involved in the pathogenesis of NSCLC.

肺癌是起源于支气管粘膜或腺体的恶性肿瘤，是发病率和死亡率增长最快、对人群健康和生命威胁最大的恶性肿瘤之一，根据统计肺癌的年发病人数及年死亡人数均居全球肿瘤首位^[1]^。肺癌的发病机制尚未完全明确，可能与吸烟、职业、环境接触、电离辐射、既往肺部慢性感染、遗传及大气污染等因素有关，肺癌的早期症状不明显，甚至可无任何不适，因此研究肺癌的发病机制及早期诊断对肺癌的治疗及预后至关重要。长链非编码RNA（long noncoding RNA, lncRNA）是一类转录本长度超过200 nt的RNA分子，它们并不编码蛋白，而是在表观遗传、转录以及转录后等多种层面上调控基因的表达水平^[2, 3]^，研究证实lncRNA广泛参与了细胞增殖、分化及凋亡等过程，在多种疾病病理过程中发挥重要作用^[4]^，与肿瘤的发病、转移、预后及治疗密切相关^[5, 6]^。HOX转录反义RNA（HOX transcript antisense RNA, HOTAIR）是一种新近发现具有反式转录调控作用的lncRNA^[7]^，国内外研究^[8-12]^发现HOTAIR在肺癌组织中高表达，且与肺癌的临床分期、淋巴结转移及预后有关。为了进一步研究HOTAIR在肺癌诊断及预后中的作用，本实验采用实时定量聚合酶链反应（polymerase chain reaction, PCR）检测非小细胞肺癌（non-small cell lung cancer, NSCLC）患者血清中HOTAIR水平，并分析其与临床病理参数之间的关系。

## 1 资料与方法

### 1.1 病例资料

选取西安市第一医院2016年1月-2016年11月期间经过组织病理确诊的NSCLC患者64例，其中男性38例，女性26例，年龄32岁-75岁，平均（56.4±10.7）岁，组织病理类型为鳞癌23例、腺癌39例、大细胞癌2例，分期采用2009年国际抗癌联盟（Union for International Cancer Control, UICC）（第7版）肺癌肿瘤-淋巴结-转移（tumor-node-metastasis, TNM）分期标准进行：Ⅰ期15例，Ⅱ期20例，Ⅲ期26例，Ⅳ期3例。64例健康体检者作为正常对照组，其中男性40例，女性24例，年龄26岁-71岁，平均（53.4±9.6）岁，本研究经过医院伦理委员会批准并征得所有研究对象的知情同意。

### 1.2 样本采集

所有患者均在进行手术、化疗或放疗前进行采样，空腹无菌采取静脉血5.0 mL，放置30 min后1, 200 g离心10 min，吸取上清至离心管中，4 ℃ 12, 000 g再离心10 min后分离上清至新的离心管中，取250 μL血清加入750 μL TRIzol^®^ LS试剂后-80 ℃保存待用。

### 1.3 总RNA提取及cDNA第一链合成

参照文献^[13]^严格按照TRIzol^®^ LS试剂（美国Invitrogen公司）说明书进行血清总RNA提取，提取的RNA溶于DEPC处理的水中。采用逆转录试剂盒（美国Thermo Scientific公司）进行cDNA第一链合成，具体如下：3 μg总RNA、5 μg/μL Oligo(dT)1 μL温和混合后70 ℃孵育5 min，冰上冷却，短暂离心收集上清；依次加入5×反应缓冲液4 μL、20 U/μL RNase抑制剂1 μL及10 mmol/L dNTP 2 μL 37 ℃温和混合后孵育5 min；加入200 U/μL M-MuLV逆转录酶1 μL，42 ℃ 60 min、70 ℃ 10 min，冰上冷却终止反应；合成的cDNA第一链在-20 ℃保存待用。

### 1.4 实时定量PCR检测

根据Genebank中HOTAIR及内参GAPDH的序列，利用Primer 5.0软件设计引物，具体序列如下：HOTAIR上游引物5'-GGCAGCACAGAGCAACTCTA-3'，下游引物5'-GCAGGGTCCCACTGCATAAT-3'，扩增产物大小125 bp；GAPDH上游引物5'-ACTAGGCGCTCACTGTTCTCT-3'，下游引物5'-GTTGACTCCGACCTTCACCT-3'，扩增产物大小83 bp，由上海生工生物技术科技有限公司合成引物。利用SYBR^®^ Premix Ex Taq^TM^ Ⅱ（大连宝生物工程有限公司）进行PCR扩增，20 μL反应体系包括10 μL SYBR^®^ Premix Ex TaqTM Ⅱ（2×）、模板3.0 μL、上下游引物（10 μM）各1.0 μL、ddH_2_O 5.0 μL，PCR反应条件：95 ℃预变性30 s，95 ℃ 10 s、60 ℃ 30 s共35个循环。扩增结束后得到Ct值，采用2^-△△Ct^法计算血清中HOTAIR的表达水平。

### 1.5 统计学方法

所有数据均采用SPSS 16.0进行统计学处理，计量资料采用均数±标准差（Mean±SD）表示，组间比较采用*t*检验，以*P* < 0.05为差异有统计学意义。

## 2 结果

### 2.1 HOTAIR在NSCLC患者血清中的水平

实时定量PCR检测结果发现NSCLC患者血清中HOTAIR水平为（2.85±1.16），明显高于正常对照组（1.74±0.69），两组之间差异具有统计学意义（*t*=6.566, *P* < 0.01）。

### 2.2 NSCLC患者血清中HOTAIR水平与临床病理参数之间的关系

为了进一步研究HOTAIR在NSCLC发生、发展中的作用，本研究统计分析了肺癌血清中HOTAIR水平与临床病理参数之间的关系，结果发现血清中HOTAIR水平与患者性别、年龄、吸烟、分化程度及病理类型均无明显相关（*P* > 0.05）。而肿瘤大小 > 3 cm的肺癌患者血清中HOTAIR水平明显高于肿瘤大小≤3 cm患者，Ⅲ期+Ⅳ期肺癌患者血清中HOTAIR水平明显高于Ⅰ期+Ⅱ期患者，有淋巴转移的肺癌患者血清中HOTAIR水平明显高于无淋巴转移的患者，差异均具有统计学意义（*P* < 0.05），结果见表 1。

**1:** NSCLC患者血清中HOTAIR水平与临床病理参数的关系 The relationships between the serum levels of HOTAIR and clinical pathological parameters in the patients with NSCLC

Clinical pathological parameters	Cases (*n*)	Relative expression of HOTAIR（Mean±SD）	*t*	*P*
Age (yr)			0.775	0.221
> 65	17	3.04±1.28		
≤65	47	2.78±1.15		
Gender			0.333	0.739
Male	38	2.81±1.15		
Female	26	2.91±1.22		
Smoking			0.531	0.298
Yes	30	2.94±1.24		
No	34	2.78±1.17		
Tumor size			2.433	0.009
> 3 cm	36	3.17±1.33		
≤3 cm	28	2.42±1.07		
TNM stage			2.539	0.007
Ⅰ+Ⅱ	35	2.49±1.13		
Ⅲ+Ⅳ	29	3.28±1.36		
Differentiation			1.029	0.154
Well/Moderate	40	2.73±1.17		
Poor	24	3.05±1.26		
Lymph node metastasis			2.980	0.002
Negative	27	2.34±0.97		
Positive	37	3.22±1.29		
Histology^a^				
Squamous cell carcinoma	23	2.67±1.18	0.896	0.187
Adencarcinoma	39	2.96±1.25		
^a^: 2 cases of large cell carcinoma were not counted. NSCLC: non-small cell lung cancer; HOTAIR: HOX antisense RNA. TNM: tumor-node-metastasis.				

## 3 讨论

非编码RNA（non-coding RNA）是指不编码蛋白质的RNA，其中包括rRNA、tRNA、snRNA、snoRNA、microRNA、lncRNA及circRNA。由于lncRNA不编码蛋白质，最初被认为是基因组转录的“噪音”，不具有生物学功能。但随着RNA测序、芯片等分子生物学技术和生物信息学的不断发展，越来越多的研究^[4-6]^证实lncRNA具有复杂的生物学功能，在多个层面上参与细胞分化和个体发育调控，并与多种疾病尤其是恶性肿瘤的发生发展密切相关。国内外学者研究^[14-16]^发现MALAT1、ANRIL、H19、MEG3、GAS5及CCAT2等多种lncRNAs在肺癌中异常表达，与肺癌的发病、诊断、治疗及预后密切相关。HOTAIR是Rinn等^[7]^发现的第一个具有反式转录调控作用的lncRNA，人HOTAIR基因定位于12q13区域，基因（RefSeq NR_003716）全长2, 364 bp，由1个长外显子和5个短外显子组成，HOTAIR主要是通过5'结构域和3'结构域募集复杂蛋白复合体来调控靶基因的表达。多项研究发现HOTAIR不仅在乳腺癌、食管癌、胃癌、肝癌、结肠癌、肾癌、宫颈癌、卵巢癌、胰腺癌、肺癌等实体瘤中高表达^[17, 18]^，而且在淋巴瘤、急性白血病等血液系统恶性肿瘤中也高表达^[19, 20]^。国内外学者^[8-12, 21]^研究发现NSCLC患者肿瘤组织中的HOTAIR的表达水平明显高于正常肺组织，细胞实验证实过表达HOTAIR可以显著促进A549细胞的迁移和侵袭，且与顺铂化疗耐药有关。由于lncRNA具有疾病特异性和组织特异性、稳定性高等特点，在心血管疾病、肿瘤患者外周循环中可以检测到lncRNA，有可能成为疾病诊断、预后的标志物^[22, 23]^。本研究采用实时定量PCR检测NSCLC患者血清中HOTAIR的水平，结果发现NSCLC患者血清中HOTAIR水平明显高于正常对照组（*P* < 0.01），这与国内外学者在肺癌组织中的研究结果一致^[8-12]^，提示HOTAIR在NSCLC病理过程中发挥了重要作用。

本研究进一步分析了NSCLC患者血清中HOTAIR水平与临床病理参数之间的关系，结果发现肿瘤大小 > 3 cm的肺癌患者血清中HOTAIR水平明显高于肿瘤大小≤3 cm患者，Ⅲ期+Ⅳ期肺癌患者血清中HOTAIR水平明显高于Ⅰ期+Ⅱ期患者，提示HOTAIR与TNM分期及肿瘤负荷有关，与国内外学者研究结果^[8-12]^一致，本研究还发现Ⅰ期+Ⅱ期患者血清中HOTAIR水平仍然明显高于正常对照组（*P* < 0.01），随着研究的不断深入HOTAIR有可能成为肺癌的早期诊断指标之一。同时研究结果还发现有淋巴转移的肺癌患者血清中HOTAIR水平明显高于无淋巴转移的患者，有研究表明过表达HOTAIR可以显著促进肺癌细胞A549的迁移和侵袭^[11, 24]^，提示HOTAIR参与了肺癌的侵袭及转移过程。本研究结果显示肺癌患者血清中HOTAIR水平与患者性别、年龄、吸烟、分化程度及病理类型均无明显相关，但张泽雨^[10]^等发现NSCLC患者肿瘤组织中HOTAIR的水平与肿瘤的分化程度明显相关，这可能与病例的选择有关，有待于扩大样本量进行进一步研究。

综上所述，本研究发现NSCLC患者血清中HOTAIR水平升高，且与肿瘤大小、TNM分期及淋巴转移有关，提示HOTAIR与肺癌的发生发展及转移有关，但其具体作用机制尚不明确。由于肺癌的临床表现比较复杂，早期症状不明显，甚至可无任何不适，因此肺癌的早期诊断尤为重要，随着对HOTAIR研究的不断深入，可能为肺癌的早期诊断及治疗提供新的靶点。
